# 

**Published:** 1866-05

**Authors:** 


### Books and Pamphlets Received.

The Transactions of the American Medical Association. Vol. xvi. Philadelphia: Printed for the Association.

Asiatic Cholera. By F. A. Burrall, M. D. New York: Wm. Wood & Co.

Biographical Sketches of Distinguished Living New York Surgeons. By Samuel W. Francis, A. M., M. D., Fellow of the New York Academy of Medicine. Re-printed from the Philadelphia Medical and Surgical Reporter New York. Published by John Bradburn, 1866.

Recent Advances in Ophthalmic Science. The Boylston Prize Essay for 1865. By Henry W. Williams, M. D., Ophthalmic Surgeon to the City Hospital, Boston, University Lecturer on Ophthalmic Surgery in Harvard University, member of the American Ophthalmological Society, etc., etc. Boston: Ticknor & Fields, 1866.

On the Mechanical Treatment of Chronic Inflammation of the Joints of the Lower Extremities, with a description of some new apparatus for producing extension of the Knee and Ankle-joints. By Lewis A. Sayre, M. D., Surgeon Bellevue Hospital, Professor of Orthopædic Surgery Bellevue Hospital Medical College, Resident Physician of the City of New York, Member of the American Medical Association, New York State Medical Society, New York Academy of Medicine, New York Pathological Society, etc., etc. Extracted from the Transactions of the American Medical Association. Philadelphia, 1865.

Descriptive Catalogue of Fluid and Solid Extracts in Vacuo, also Concentrations and Officinal Pills, prepared by Henry Thayer & Company, with formulas and receipts, 1866.

Report of the Board of Trustees of the Massachusetts General Hospital for the year 1865.

Diarrhœa and Cholera, their Origin, Proximate Cause and Cure through the agency of the Nervous System, by means of Ice. By John Chapman, M. D., M. R. C. P., M. R. C. S. Re-printed with additions from the Medical Times and Gazette of July 20th, 1865. Philadelphia: J. B. Lippencott & Co., 1866.

The Application of Sutures to Bone, in recent Gun-shot Fractures with cases; also remarks on their similar use in some other Fractures and Operations. By Benjamin Howard, M. D., Fellow of the College of Physicians and Surgeons, New York; late Assistant Surgeon, Regular Forces, U. S. Army.

Twenty-Third Annual Meeting of the Managers of the State Lunatic Asylum for the year 1865,

Prospectus of Southern Medical and Surgical Journal, Augusta, Ga. Joseph Jones, M. D., Editor.

Prospectus of Nashville (Tenn.) Journal of Medicine and Surgery. W. K. Bowling, M. D., Editor.

Prospectus of the New York Eclectic Medical Review. James A. Hershall, M. D., Editor.

